# Combining Threshold, Thurstonian and Classical Linear Models in Horse Genetic Evaluations for Endurance Competitions

**DOI:** 10.3390/ani10061075

**Published:** 2020-06-22

**Authors:** Isabel Cervantes, Juan Pablo Gutiérrez, Silvia García-Ballesteros, Luis Varona

**Affiliations:** 1 Departamento de Producción Animal, Universidad Complutense de Madrid, Avda. Puerta de Hierro s/n, E-28040 Madrid, Spain; icervantes@vet.ucm.es; 2 Departamento de Mejora Genética Animal, Instituto Nacional de Investigación y Tecnología Agraria y Alimentaria, E-28040 Madrid, Spain; silvia.garciab@inia.es; 3 Departamento de Anatomía, Embriología y Genética Animal, Instituto Agroalimentario de Aragón (IA2), Universidad de Zaragoza, E-50013 Zaragoza, Spain; lvarona@unizar.es

**Keywords:** race, time, rank, placing, genetic evaluation, horse

## Abstract

**Simple Summary:**

Endurance competitions are carried out across the countryside worldwide, where the adapted physical and metabolic conditions of the horses are essential to satisfactorily end the competition. Horse breeding programs usually use the rank and time to improve the performance of horses in competitions. It is also relevant to analyse the placing trait i.e., whether or not a horse finishes the race. The discontinuous nature of rank and placing traits require special methodologies to deal with them. Here we have used 6135 endurance records from 1419 horses, with a pedigree containing 10,868 animals, to develop a multitrait model with a new free software tool (GIBBSTHUR). The obtained results suggest that it is possible to ignore the race time and use rank and placing to perform the genetic evaluation in endurance horse populations.

**Abstract:**

The racing time and rank at finish traits are commonly used for endurance horse breeding programs as a measure of their performance. Even so, given the nature of endurance competitions, many horses do not finish the race. However, the exclusion of non placed horses from the dataset could have an influence on the prediction of individual breeding values. The objective of the present paper was to develop a multitrait model including race time (T), rank (R) and placing (P), with different methodologies, to improve the genetic evaluation in endurance competitions in Spain. The database contained 6135 records from 1419 horses, with 35% of the records not placed. Horse pedigree included 10868 animals, with 52% Arab Horses. All models included gender, age and race effect as systematic effects and combined different random effects beside the animal and residual effects: rider, permanent environmental effect, and interaction horse-rider. The kilometers per race was included as a covariate for T. Heritabilities were estimated as moderately low, ranging from 0.06 to 0.14 for T, 0.09 to 0.15 for P, and 0.07 to 0.17 for R, depending on the model. T and R appeared mostly as inverse measures of the same trait due to their high genetic correlation, suggesting that T can be ignored in future genetic evaluations. P was the most independent trait from the genetic correlations. The possibility of simultaneously processing the threshold, Thurstonian and continuous traits has opened new opportunities for genetic evaluation in horse populations, and much more practical genetic evaluations can be done to help a proper genetic selection.

## 1. Introduction

Horse breeding programs present particularities that differ from other classical livestock breeding programs, which require breeders to make an additional effort to develop useful tools, specifically for horses. Among horse breeding programs, rank endurance competitions are an example of the difficulties genetic evaluation can present. For example, as in many other performance competitions, often only the final position of the horse is registered, thus complicating the genetic evaluation process. By definition, if the rank trait is analyzed as if it was a continuous variable, it implies a uniform distribution within a race if the rank is expressed over the number of participants. Unfortunately, the reality is that the number of participants varies and sometimes not all of the animals are available, thus leading to a right skewed distribution [1]. Fortunately, however, the symmetric shape of the distribution of the rank, transformed to account for the number of participants, revealed that there was not a preference in the register of best positions [1]. Anyway, whether distributions are uniform or skewed, they are a major concern for genetic evaluation. This concern was investigated by other authors [1,2,3] with each author arriving at very interesting solutions. The use of threshold models [4] is widely applied in many other species and traits, and has been the mainstay in these types of traits [5,6]. Under this model, it is assumed that an underlying non-observable variable exists, defining the different categories of the categorical trait if this underlying variable exceeds a particular threshold value. One major concern of these models is the number of thresholds that have to be defined. This makes it necessary to gather all individual rankings after an arbitrary position to belong to the same group category, which artificially introduces an additional source of error. In addition, probably the main concern of the ranking trait is that it is highly conditioned by the level of competitors in the same event. A recent and accepted alternative solution was the use of Thurstonian models [7] that were applied in horse populations [6,8]. These Thurstonian models have not been extensively used in a multitrait analysis combined with other traits, because, until now, no software was available to carry out genetic evaluations under this methodology. An alternative computation would be to transform the rank records to a Gaussian distribution by matching the requirements of well developed mixed model classical methodology [1]. However, with this approach, the levels of competitors participating in the same race were not considered in the transformed variable.

Endurance competitions are long distance races that vary from 40 km to 160 km per day. The competition can reach more kilometers, but in this case several days are needed to finish the race. This type of competition is carried out across the countryside, where the adapted physical and metabolic conditions of the horses are essential to finish the race. Horses can be excluded after veterinary controls when they exceed a determined heart rate or certain metabolic parameters or lameness during the endurance exercise. A good animal for endurance performance would be a strong animal achieving a good position, but one that is also veterinary protected. Obtaining good positions and keeping in the race might be opposed features. The Spanish Arab Horse Breeder Association (AECCA) is currently developing a breeding program aimed at improving endurance performance. This selection objective is common in other crossbreeds such as Anglo-Arab, Hispano-Arab and Caballo de Deporte Español in Spain. However, endurance competitions are open to other breeds with very different breeding objectives.

The race time and the ranking at the finishing line are traits commonly used for endurance horse breeding programs as a measure of their performance. Given that race time or speed is highly correlated with the ranking trait, they are frequently used in the genetic evaluations as alternatives, and they also have a more suitable Gaussian distribution [9,10]. An additional difficulty with endurance competition horses is the disqualification of some animals for health reasons, to protect them from demanding riders. Obviously, an animal that does not finish races on a regular basis is not very attractive to breeders. Disqualified horses are sometimes listed as finishing the race and listed at the end of the race record, and maybe assigned an additional position [6]. For the purpose of genetic evaluation, these horses can be removed from the dataset and placed in a two trait category (placed or not placed). This two trait category, called placing, can then be analyzed as a different threshold trait [10]. Placing refers to those animals finishing the race with a defined specific position.

The Thurstonian methodology has proved to be the most suitable method to study genetic evaluation of rank in endurance horse competitions in Spain; it was validated using a univariate model for rank [6]. The models would perform better if placing was removed from the dataset and analyzed under a threshold model methodology. The relationship between both (rank and placing), and the race time is also a parameter of interest, but the time trait is analyzed under the classical linear model methodology. Thus, the objective of the present study was to estimate the genetic parameters of these three traits, race time (T), rank (R) and placing (P) by applying for the first time a multitrait animal model that used different methodologies for each trait (threshold, Thurstonian and classical models). To shed light on the best way to genetically select animals for this equestrian discipline, the models fitted genetic groups from different origins, and also several random additional effects besides additive genetic and residuals.

## 2. Materials and Methods

**Figure 1:**
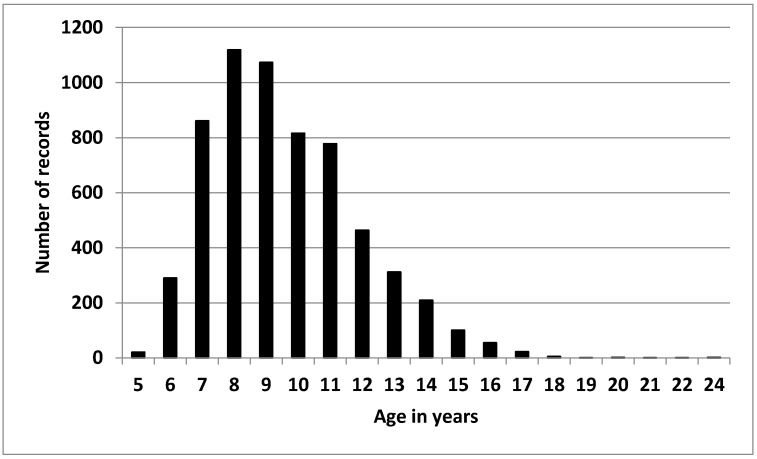
Distribution of records regarding age in years.

The total data set consisted of 6135 records from 1419 horses (553 males, 605 females and 280 geldings, 19 of them performing both as male first and as gelding after castration in different races), aged between 5 and 24 years (Figure 1). Most of them were Arab horses (70.6%) and the rest were Anglo-Arab (19.8%), Caballo de Deporte Español (6.3%) and other breeds (3.2%, Hispano-Arab, Pura Raza Español, Thoroughbred and Trotters). The records were collected by the Spanish Royal Equestrian Federation in competitions that followed the Fédération Equestre Internationale (FEI) rules. The dataset contained 1275 endurance races held between 2000 and 2018. The number of races available per year ranged between 8 and 114. Most of them, 88%, were held in Spain, 8% in France and the rest in other countries (Portugal, Italy, Great Britain and Emirates). The race time was available for 2997 records, accounting for 76% of the horses that finished the race. Of the total number of events, 8% had more than 12 participants, and the average number of records per event was 4.8. A description of the data set is shown in Table 1. The number of records per horse ranged between 1 and 32, with an average of 4.3; a total of 2172 of the records were for disqualified horses.

The number of different riders in the data set was 1018 and the average number of different riders riding the same horse was 1.7. In total, 45% of the horses were ridden by two different riders or more. In addition, the average number of different horses ridden by a rider was 2.5, and 50.8% of riders rode at least two different horses. The rider–horse interaction or match effect had an average of 2.4 records across 2550 levels.

Pedigree was traced back by using the information from the corresponding pure Studbooks. Pedigree information for genetic evaluation totaled 10868 animals: 52% Arab Horses, 18% Thoroughbred, 12% Pura Raza Español, 10% Anglo-Arab, 2% Caballo de Deporte Español and 6% other breeds. This pedigree was built by completing the genealogy of participant animals until the maximum known generation. In order to measure the pedigree completeness, the equivalent complete generations were computed for each participant. The mean of equivalent complete generations for participating horses was 7.1 for Arab Horses, 3.5 for Thoroughbred, 5.9 for Anglo-Arab and 3.8 for Caballo de Deporte Español. This parameter was computed with ENDOG4.8 [11].

The following traits were jointly analyzed: Race Time in hours (T); Placing, with two thresholds, 1 not placed and 2 placed (P); and Rank race (R) traits. The unplaced horses were considered as missing values for T and R traits. The genetic parameters were estimated for these three traits using a different methodology for each of them. A Bayesian procedure was applied by fitting a classical mixed linear method for T, a threshold method for P, and a Thurstonian method for R.

The general mixed model equation applied across methods including all the possible random effects was:

**I = Xb + Zu + Wp + Qr + Nm + e** (1)

where **I** is the phenotypic record for race time or the liability for placing and rank, **X** is the incidence matrix of systematic effects, **Z** the incidence matrix of animal genetic effects, **W** the incidence matrix of permanent environmental effects, **Q** the incidence matrix of the rider effect, **N** the incidence matrix of match effects, **b** the vector of systematic effects, **u** the vector of direct animal genetic effects, **p** the vector of permanent environmental effects, **r** the vector of rider effects, **m** the vector of match effects and **e** the vector of residuals. In addition to the complete model shown in the equation above, all of the model combinations, ignoring one or two effects from **p**, **r** and **m**, were also fitted.

Three different methodologies were jointly considered depending on the trait:

### 2.1. Classical Linear Model Fitted for T

For the T trait, vector **I** contained the race time of the horses in hours as a continuous variable. However, vector **I** was assumed to be the liabilities for an underlying variable, as described below for the P and R traits. Note that for the T trait, the lower the performance, the better the horse.

### 2.2. Threshold Model Fitted for P

Threshold model methodology, also called probit [4,12,13] was used to analyze the P trait. It would theoretically fit the discrete probabilistic nature of the data better. The trait was codified as: 1 for unqualified horses and 2 for horses ending the race. Thus, unlike T, higher values would explain better performances. Under this model, it is assumed that there is an underlying non observable variable defining the different categories of the categorical trait if this underlying variable exceeds or doesn’t exceed a particular threshold value. Under the Gibbs sampler scheme, the underlying variable associated with each observed phenotype is sampled from a truncated Gaussian distribution, with mean and variance defined by the conditional expectation and variance given to the remaining unknowns in the model. Being unobservable, the residual variance of the liability is set to 1.

### 2.3. Thurstonian Model Fitted for R

A Thurstonian model was fitted for R. Under this methodology, ranks were renumbered for the animals registered for each race. The statistical analysis was performed with a specific Bayesian approach designed for Thurstonian models suggested by Gianola and Simianer [7]. A Thurstonian model can include and evaluate the competitive level of horses in races, which is transformed via a data augmentation step within a Gibbs Sampler for rank trait. The vector y in the model above would be filled by the liabilities of each horse participating in each race. As in threshold models, liabilities of the Thurstonian model were not observable and it was assumed that $\sigma_{e}^{2}$ = 1.

The liability of the race winner was set to 0. The liability for any other horse n in a race was calculated by using the following truncated Gaussian distribution (*TN*) as:

(2) $$l_{n}\sim {TN}_{\left(l_{n-1},l_{n+1}\right)}(\mu_{n},\sigma_{n}^{2})$$

where: *n* − 1 and *n* + 1 for the competing horses in the respective previous and next qualifying positions in a race; *μ_n_* and $\sigma_{n}^{2}$ are the conditional expectation and variance of the liability given all the remaining effects of the model, including systematic and random effects for all traits and residual variances and covariances. Liabilities for other horses, other than the winner, are more negative the higher they are in the position. Therefore, for R, the bigger the liability, the better the performance; the same is true for P, but not for T.

All models included gender (males, females and gelding), age (ten levels for age, from 6 or less years to 15 or more) and race event as systematic effects. Race effect had different levels according to the traits because race time was not recorded in all races. This race effect was included as the even effect to account for all of the common environmental circumstances of the horses competing together and correcting their differences between events, including class and difficulty of the competition of the race, and also the mean level of competitors, as non random participation occurs. All horses qualified in some races showed: a total of 731 levels were fitted for T, 797 for P and 1275 for R. Distance in km of the race was also fitted as a covariate for the T trait, with a range from 60 to 160 km.

All of the models accounted for ten genetic groups according to the origin of the founder. The use of genetic groups is indicated when there are different populations of origin with presumably different means, as is the case in [14]. The genetic groups were Arab Horse (the number of founders in the group: 738), Pura Raza Español (241), Thoroughbred (848), Anglo-Arab (146), Hispano-Arab (20), Sport Horse (33), Selle Français (39), German origin (74), Belgian origin (8) and others (154).

A global model including all the random effects was fitted, but also it excluded one or two of the additional random effects besides additive genetic and residual, which were fitted.

We used the GIBBSTHUR software [15] that is a modified version of the TM (Threshold Model) software [16] to include rank traits under a Thurstonian approach.

Total chain lengths of 2,000,000 samples for each analysis were defined, with a burn-in period of an additional 100,000 and a thinning interval of 100.

## 3. Results

The means and standard deviations of the marginal posterior distributions of heritabilities are given in Table 2. In addition, in Table 2 are the variances over phenotypic ratios of rider, match and permanent environmental effects and correlation between traits and for the corresponding effects for the whole and the reduced models, ignoring some random effects. The table gathers all the estimated parameters by rows with additional ones for their standard deviation, and also gathers all of the fitted models by columns specifying the random effects included in each. Within each crossed subgroup of parameters, there are the ratio estimates in the diagonals in bold, with correlation between traits off-diagonals.

Depending on the model being considered, heritabilities were estimated as rather low, ranging from 0.06 to 0.14 for T, 0.09 to 0.15 for P, and 0.07 to 0.17 for R. Regarding genetic and other correlations between random effects, T and R mostly appeared as inverse measures of the same trait since these correlations ranged from −0.97 to −0.99 across models and effects. P was the most independent trait since the genetic correlations of P with T ranged from −0.03 to −0.16 and of P with R from 0.07 to 0.20. This pattern was similar across effects except for the match effect when correlations were from −0.39 to −0.44 with T, and from 0.42 to 0.49 with R. This result shows that a good horse-rider match affects both qualifying for a race and in a good position. This favorable combination did not happen when the rider or the horse effects were analyzed independently.

When fitting all the random effects, the interaction between the horse and the rider, known as the match effect (0.06 for T, 0.10 for P and 0.08 for R), had a similar magnitude of importance to the heritability component for all the three traits (0.06 for T, 0.09 for P and 0.07 for R). The permanent effect (0.06 for T, 0.03 for P and 0.08 for R) also had a similar importance for T and R, but was insignificant for P. The rider effect (0.12 for T, 0.04 for P, and 0.17 for R) was the most important among the random effects for T and R, but again mostly insignificant for P, for which the match effect was the most important.

The standard deviations of the estimated parameters were low for heritabilities and for the ratios of additional random effects (0.02 to 0.04) and moderate for correlations (0.18 to 0.28), but larger for permanent environmental effect (0.28 to 0.39).

Regarding reduced models, excluding one or two random effects (rider, permanent environmental and match effect), they always increased the heritability of the traits. Excluding the permanent environmental effect increased the heritability of both T and R by 49%, but only 19% for P, and excluding the rider effect increased the heritability of T by 39%, R by 42%, but only 9% for P. However, excluding the match effect barely increased the heritability of both T and R with 2%, but P increased by 28%.

When two effects were simultaneously excluded, heritability became even higher. For P, the model fitting only the match effect performed similarly to the model that fitted the rider effect and the permanent effect as two separate effects; both models had similar heritability estimates showing that the match effect incorporates the sum of both the rider effect model and the permanent effect model. There was a different pattern for T and R, heritability estimates were more than twice those of the complete model for both traits when only match effect was included, showing a confusion between rider and permanent effect with the additive genetic effect. Keeping the rider or permanent effect in the model obtained lower heritabilities than keeping the match effect, but absorbing the residual, which is an important part of the non fitted random effects.

**Figure 2:**
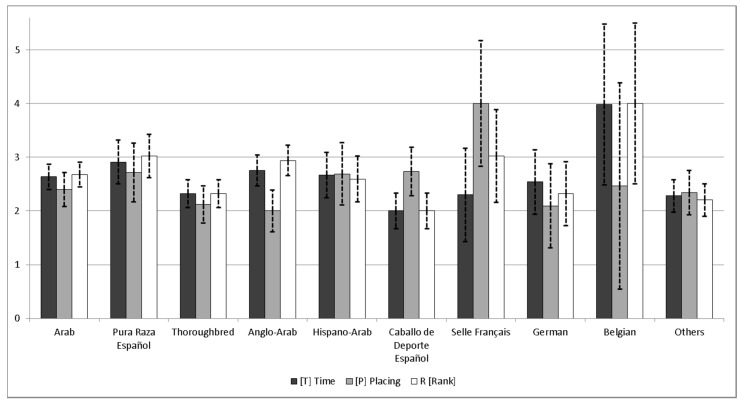
Means and standard deviations of the marginal posterior distributions for the genetic groups, transformed to positive scale, with the minimum fixed to 2.

Solutions for the complete model, including all the random effects, were analyzed to make inferences from effects. Figure 2 shows the estimates of the genetic groups as the mean and the standard deviation of their marginal posterior distributions for the analyzed three traits; the sign for T was changed to a positive value and with a minimum value set arbitrarily to 2. There were no significant differences among them across traits, but Belgian breed horses performed the best for T and R, and Selle Français for P. The Caballo de Deporte Español performed the worst for T and R, and the Anglo-Arab for P. It has to be noted that, for most of the breeds, there were no participating animals having a performance, but only their crosses with other breeds, thus these solutions are mainly showing the combinatory ability of the breed. Moreover, the low number of individuals in some genetic groups could also create a bias in the results and high standard deviations. After taking a closer look at the genetic groups with more information—Arab, Pura Raza Español, Thoroughbred and Anglo-Arab—Pura Raza Español horses showed better values in all traits. The Arab horse showed intermediate values, achieving better results for all traits than Thoroughbreds and better results for P than Anglo-Arab horses. Pura Raza Español is not an endurance breed, but is crossed to produce Hispano-Arab horses for endurance races.

**Figure 3:**
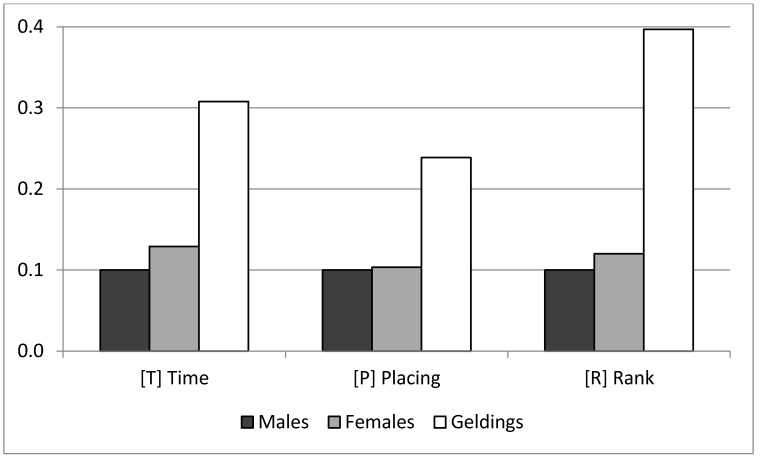
Means of the marginal posterior distributions for the levels of the sex effect for Race Time, Placing and Rank traits, transformed to positive scale, with the minimum fixed to 0.1.

The estimate of the corresponding covariate for distance showed that horses took on average 69.4 s per additional kilometer. Regarding the gender influence, Figure 3 shows the mean and standard deviations of the marginal posterior distribution for the sex levels effect; this effect was transformed to a positive scale with the minimum value arbitrarily fixed to 0.1. Geldings performed better than the other horses for the three traits, with no relevant difference between males and females. In the case of T, geldings saved roughly about 12 min per race compared with males and females.

**Figure 4:**
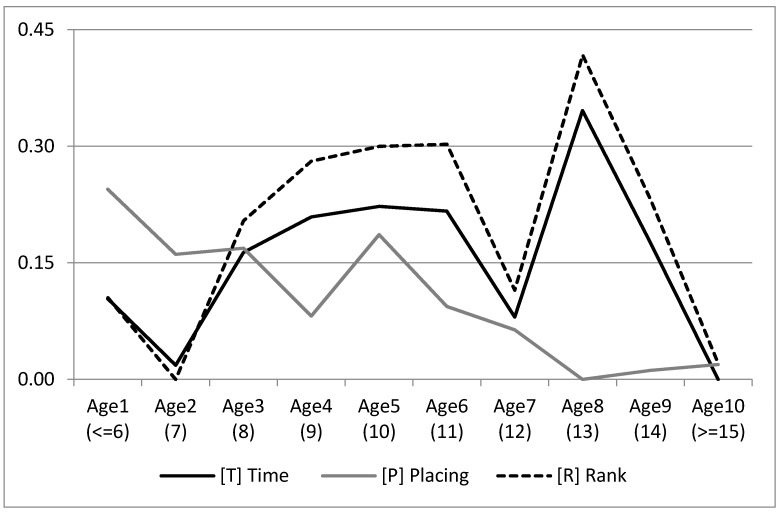
Means of the marginal posterior distributions for the levels of age for Race Time, Placing and Rank traits, transformed to positive scale, with the minimum set to 0.

Figure 4 shows the marginal posterior distribution means for the levels of age of the three traits, with the minimum set to 0. P tended to worsen with age. T and R had a minimum age value of seven years, then with a quadratic improvement until 11 years, after which the trend become irregular, probably because of fewer records for these levels.

## 4. Discussion

Analyzing the genetic evaluation from horse competitions is an issue that presents additional particularities for endurance races. The goal for genetic evaluations is to breed animals with special abilities to win races or to score relevant positions in races. However, the direct use of such position scores does not always provide the best information to genetically select animals [17]. Apart from the categorical nature of R, there are other issues of concern such as: the theoretical uniform and consequently non-Gaussian distribution; or the skewness consequence of the different number of participants, in which there is always a winner, but not always a high number of positions; or there is also a preference to register animals with outstanding performances [1]. In addition, R also depends on the level of competitors participating in the same race. Thus, modeling R is a challenge, this trait having been essayed as if it was a continuous trait; or applying threshold models; or fitting Thurstonian model methodologies [6,17]; or transforming it to match a Gaussian distribution shape [1,18]. García-Ballesteros et al. [6] concluded that the Thurstonian model was the most suitable to analyze R when compared to threshold or continuous methodologies. Another alternative would be to use a continuous trait that is supposed to be highly related with the ranking, i.e., T, and has been often essayed as an alternative, mainly in Thoroughbred and trotter races [9,19,20,21].

Another particularity of endurance races is the veterinary control that can disqualify horses: 35% of the horses were disqualified because of heart rate, metabolic problems, limb problems such as lameness, and other veterinary reasons (e.g., skin problems) [22]. This disqualification percentage was similar to the percentage found in other studies [23]. The ideal horse would combine high speed (low race time) with enough robustness to satisfy the veterinary controls. Therefore, the information about placing or not is essential to account for this issue of disqualification. To deal with this situation, unqualified horses have sometimes been considered as performing behind the last horse to perform a genetic evaluation of R, thus combining in this way a placing into the usual trait under selection [6]. However, moving away from this combined trait definition, P has been better fitted as a different threshold trait, with finishing the race as one category, and unqualified in another category, leading to performing a two traits model [10].

A study was done to analyze race time, ranking, and placing simultaneously in a multitrait animal model considering the last two traits as threshold traits. The ranking trait involved eight different categories, grouping all individuals ranking higher than six within the 7th category, and grouping the non placed individuals within the 8th category [6,24]. In this previous study, race time was excluded as an alternative to ranking because the estimated genetic correlation between them was 0.62. This genetic correlation was lower than expected, partly because of the artificial grouping for the last two categories of the ranking trait. In fact, under univariate analyses, estimated heritability for ranking moved from 0.11 to 0.27 when records from non placed animals were removed. An additional concern was that information about placing would also be part of the ranking trait.

García-Ballesteros et al. [6] demonstrated that Thurstonian models were better for ranking traits than threshold and linear models, even accepting that there can be any distances between consecutive participants in the underlying working variable. Here, the challenge was the joint analysis of Thurstonian, threshold and continuous traits carried out for genetic parameters estimation for the first time, eliminating the difficulties of this equestrian sport. In the present study, the most striking result was the relationship between T and R. Negative correlations between these two traits have to be understood as a favorable relationship according to the description of the traits in the corresponding section. All the correlations across effects and models were lower than −0.96, suggesting that both traits address the same conceptual performance, and one of them can be ignored. In this respect, R would be kept in future analyses instead of T for several reasons. Firstly, because it has about 25% higher heritability. Secondly, because R was performed for all of the horses, while T was only available for 49% of the records. Finally, because in this particular dataset, this T trait was not always reliable and many records were lost. Solving a multitrait model using two discrete variables such as R and P, and adding a continuous trait, opens up the option to use a morpho-fuctional trait [25] or physiological parameters such as heart rate [26] to analyze with R and P. These variables can be identified in young animals and, given that conformation and physiological traits are an early indicator of sportive performance, this can lead to more efficient breeding improvements [25].

Fitting different additional random effects besides residual and additive genetic effects has always been a topic of debate [6,27]. The permanent environmental effect, the rider effect, and the interaction between rider and horse (match effect) [28,29,30], can be included in the models, fitting some or all the effects together [6]. The rider-horse interaction, the match effect, would explain the relationship, cooperation and communication between horse and rider. The match effect is influenced by the level of experience and behavior of both rider and horse performing together, and measures the different behavior traits a horse has with specific riders [1]. García-Ballesteros et al. [6] reported that a model including all the random effects was the best option for this endurance competition population. The complete model was better at showing the relative importance of the different random effects. The most important was the rider for T and R (0.12 and 0.17, respectively). However, the placing did not mainly depend on the horse or the rider, but, because rider and horse understand each other; the match effect was the most important P trait. Basically, it seems that some riders can better interpret the fatigue signs from some horses than from others, while the same signs in other horses are well interpreted by different riders. This was likewise endorsed by watching which effects were confounded with those random effects that were ignored in the estimations. Thus, ignoring the rider or permanent effect increased the heritability of T and R by roughly 40%, but only around 10% for P. Ignoring the match effect did not affect T and R, but increased P by nearly 30%. The best option to obtain a good position in a race is to have a good rider with a good horse, and the choice of rider–horse combination is important in order to have a good chance of finishing the race.

There are still other difficulties in the genetic evaluation of endurance. One of them is that although genetically connected, the participating horses are from different breeds and crosses. This implies having to consider different origins with genetic levels; it is, therefore, advisable to use genetic groups in the models [14]. The genetic group solution (Figure 2) did not show any significant differences amongst the breeds. This shows how little importance breed origin has in crossed horses that participate in endurance races, since many of the horses defined in the groups only appear in the pedigree as parental breeds of cross horses. Given this scenario and in the light of the solutions, a model without genetic groups would be perfectly valid. In fact, although not shown here, such a model performed very similarly to the model discussed here.

The solutions for the gender effects (Figure 3) showed that for the three analyzed traits, the ideal would be to compete riding a gelding. This is something widely known and carries an important consequence for the breeding program. Since the best horses are usually discarded for reproduction because they are usually castrated, there is a need to impose the norm of freezing semen for any male before accepting it for endurance competitions.

The pattern of age solutions (Figure 4) implies two important lessons. First, the optimal age of animals to compete is nine years, remaining fairly stable up to eleven years. The other lesson arisen by the results is the need to include animals older than eleven years together in the same level of the age systematic effect for genetic evaluations. It is highly probable that only the genetically best animals keep performing for long after eleven years old, and, having a low number of records inside those effect levels, the estimation would be confounded with the age effect. The first level of the age effect performed unexpectedly higher than the second one, possibly due to the fact that those animals starting early to compete were a biased sample.

The agreement across traits for gender effect, age solution, and correlation between traits for rider–animal interaction shows that a combination of a good rider and gelding animal competing at eleven years old is best for better performance.

From all of the gathered information, genetic evaluation would be best done for P under a threshold model and for R under a Thurstonian model methodology. The development of open source modifiable software is an important advance for this purpose of genetic evaluation [15]. The challenge is now the combination of the predicted breeding values for P and R to select the animals. An alternative might be using selection indexes defined from the genetic parameters [31] that have already been proven in horses [24,25]. The Spanish Association of Arab Horse Breeders (AECCA) is currently using a combined selection index using P (40%), R (50%) and T (10%), but, in the present paper, the race time effect is left out, and a genetic index is suggested using only P and R. However, it is essential that a horse is placed to score a position, suggesting that the selection weight would have to be on P. It could also be suggested to use the classical methodology of independent culling levels [32]. The importance each breeder gives to the probability of placing can be different among breeders, thus a single index might be controversial. An alternative solution can be the use of a range of indexes, thus breeders would be free to choose. Fortunately, P and R do seem to have no unfavorable genetic correlations, and improvement can be done on one of the traits without negatively affecting the other trait.

## 5. Conclusions

Summarizing, the development of a free software application to deal simultaneously with threshold, Thurstonian and continuous traits will open a new world to the genetic evaluation of horse populations. Moreover, genetic evaluations can be done to achieve better genetic selection. This new tool was successfully applied in endurance horse competitions in Spain and the genetic parameters estimates are reported here. The selection for ranking and placing traits seem sufficient to carry out appropriate genetic selection processes, but an analysis involving continuous traits, such as conformation or physiological variables, remains feasible for future changes in selection objectives.

## Figures and Tables

**Table 1:** Structure by systematic effect and number of records of endurance races included in the database.

	Number of Animals	Number of Records
Total animals	1419	6135
Females	605	2428
Males	553	2360
Geldings	280	1347
Anglo-Arab Horses	281	1161
Caballo de Deporte Español	90	393
Arab Horses	1002	4414
Other breeds Horses	46	167
Average by event	4.8	-
Average records by horses	-	4.3
<=6 years old	78	311
7 years old	184	861
8 years old	241	1119
9 years old	239	1073
10 years old	173	816
11 years old	180	778
12 years old	121	464
13 years old	81	312
14 years old	50	210
>=15 years old	72	191
Participated at two different ages	323	-
Participated at three different ages	203	-
Participated at four different ages	109	-
Participated at least five different ages	145	-

**Table 2:** Means and standard deviations (Std Dev) of the marginal posterior distributions of heritabilities (h^2^) and variance over phenotypic ratios of rider, match and permanent (Perm) in diagonals and in bold letters, and correlations between Race Time (T), Placing (P) and Ranking (R) traits for the corresponding effects off-diagonals for the whole and the reduced models.

	Match + Rider + Perm			Match + Rider			Match + Perm			Rider + Perm			Match			Rider			Perm		
	T	P	R	T	P	R	T	P	R	T	P	R	T	P	R	T	P	R	T	P	R
h^2^	**0.06**	−0.03	−0.97	**0.09**	−0.03	−0.98	**0.08**	−0.07	−0.98	**0.06**	−0.04	−0.97	**0.14**	−0.08	−0.99	**0.12**	−0.16	−0.99	**0.09**	−0.04	−0.98
		**0.09**	0.08		**0.10**	0.07		**0.10**	0.09		**0.11**	0.11		**0.11**	0.10		**0.15**	0.20		**0.14**	0.08
			**0.07**			**0.11**			**0.10**			**0.07**			**0.17**			**0.16**			**0.11**
Std Dev	0.02	0.28	0.02	0.02	0.20	0.01	0.03	0.25	0.01	0.02	0.27	0.02	0.02	0.18	0.01	0.02	0.15	0.01	0.03	0.24	0.01
		0.02	0.27		0.02	0.19		0.02	0.24		0.03	0.26		0.02	0.17		0.02	0.14		0.03	0.23
			0.03			0.03			0.03			0.03			0.03			0.03			0.04
Match	**0.06**	−0.44	−0.97	**0.08**	−0.41	−0.98	**0.13**	−0.44	−0.99				**0.15**	−0.39	−0.99						
		**0.10**	0.49		**0.11**	0.46		**0.13**	0.47					**0.14**	0.42						
			**0.08**			**0.10**			**0.18**						**0.20**						
Std Dev	0.02	0.27	0.02	0.02	0.26	0.01	0.03	0.20	0.01				0.03	0.19	0.01						
		0.03	0.25		0.03	0.24		0.03	0.19					0.03	0.18						
			0.03			0.03			0.04						0.04						
Rider	**0.12**	−0.17	−0.99	**0.13**	−0.18	−0.99				**0.13**	−0.20	−0.99				**0.14**	−0.22	−0.99			
		**0.04**	0.17		**0.04**	0.18					**0.07**	0.21					**0.07**	0.22			
			**0.17**			**0.17**						**0.18**						**0.19**			
Std Dev	0.02	0.23	0.00	0.02	0.22	0.00				0.02	0.18	0.00				0.02	0.17	0.00			
		0.02	0.22		0.02	0.21					0.02	0.17					0.02	0.16			
			0.03			0.03						0.03						0.03			
Perm	**0.06**	−0.12	−0.97				**0.08**	−0.12	−0.98	**0.08**	−0.22	−0.98							**0.13**	−0.32	−0.99
		**0.03**	0.12					**0.03**	0.11		**0.05**	0.23								**0.06**	0.33
			**0.08**						**0.10**			**0.10**									**0.18**
Std Dev	0.02	0.39	0.02				0.03	0.38	0.02	0.03	0.34	0.01							0.03	0.28	0.01
		0.02	0.39					0.02	0.37		0.02	0.33								0.03	0.28
			0.03						0.04			0.03									0.04
